# Chicago Pathological Society

**Published:** 1884-04

**Authors:** A. H. Tagert

**Affiliations:** Secretary pro. tem.


CHICAGO PATHOLOGICAL SOCIETY.

Regular meeting, January 14. The society was called to order by the President, Dr. Angear.

The Secretary being absent from the city, Dr. Tagert was appointed Secretary *pro. tem.*

The reading of the minutes was dispensed with.

The society then listened to the reading of a paper by Dr. Angear on "Expectant Treatment."

The paper being before the society for discussion, Drs. C. J. Lewis, Clark, and several others, mentioned cases, and thoroughly discussed the subject.

The society next listened to the report of the committee for microscopical examination of specimens from a case of intestinal obstruction, reported by Dr. Skeer at the previous meeting.

Examination showed the growth to consist of round-celled sarcoma, embryonic cells; it is rarely found in the alimentary canal, and is exceedingly malignant in nature.

About fifteen members were present.

The society on motion adjourned.

A. H. TAGERT, Secretary *pro. tem.*

---

In the February number of this journal, the name of Dr. W. H. Kane King, of Mt. Sterling, Brown Co., Ill., was accidentally not appended to his exceedingly interesting letter, dated from Chihuahua, Mexico. It chanced to be omitted from the manuscript, and was, nnfortunately, not supplied by our proof-readers. We hope to present to the profession further interesting letters from the same fertile pen.

